# Antagonistic interactions peak at intermediate genetic distance in clinical and laboratory strains of *Pseudomonas aeruginosa*

**DOI:** 10.1186/1471-2180-12-40

**Published:** 2012-03-22

**Authors:** Sijmen E Schoustra, Jonathan Dench, Rola Dali, Shawn D Aaron, Rees Kassen

**Affiliations:** 1 Biology Department, University of Ottawa, 30 Marie Curie, Ottawa, ON K1N 6N5, Canada; 2 Laboratory of Genetics, Wageningen University, Droevendaalsesteeg 1, 6708 PB Wageningen, the Netherlands; 3 Ottawa Health Research Institute, 725 Parkdale Ave, Ottawa, ON K1Y 4E9, Canada

## Abstract

**Background:**

Bacteria excrete costly toxins to defend their ecological niche. The evolution of such antagonistic interactions between individuals is expected to depend on both the social environment and the strength of resource competition. Antagonism is expected to be weak among highly similar genotypes because most individuals are immune to antagonistic agents and among dissimilar genotypes because these are unlikely to be competing for the same resources and antagonism should not yield much benefit. The strength of antagonism is therefore expected to peak at intermediate genetic distance.

**Results:**

We studied the ability of laboratory strains of *Pseudomonas aeruginosa *to prevent growth of 55 different clinical *P. aeruginosa *isolates derived from cystic fibrosis patients. Genetic distance was determined using genetic fingerprints. We found that the strength of antagonism was maximal among genotypes of intermediate genetic distance and we show that genetic distance and resource use are linked.

**Conclusions:**

Our results suggest that the importance of social interactions like antagonism may be modulated by the strength of resource competition.

## Background

The production by microorganisms of proteinaceous anticompetitor toxins such as bacteriocins is often cited as a good example of an antagonistic trait [1]. The evolution of antagonistic interactions is difficult to understand because they directly harm both actor and recipient. At the level of an individual gene, this apparent paradox can be readily resolved using the framework of inclusive fitness [2], which shows that antagonistic interactions can evolve provided they produce a net benefit to actors, even if the act of antagonism itself is costly.

Bacteriocin production has the hallmark of a classic antagonistic trait that can evolve through its effects on inclusive fitness. Bacteriocins are produced by nearly all bacteria and are considered the main agents in direct antagonistic interactions between and within bacterial species [3-6]. The production of bacteriocins is costly, both in terms of the energy diverted away from other functions such as growth and, in Gram-negatives at least, because bacteriocin-producing cells release their bacteriocins through lysis and so cause cell death [5]. Importantly, cells that are isogenic to the producing strain (typically a small fraction of cells within a population produce bacteriocins at any given time) are immune to the bacteriocin, usually via an immunity protein, and so gain a benefit from bacteriocin production from clone-mates. It has also been repeatedly noted that bacteriocins are highly specific in their action, being active primarily against genetically distinct members of the same species or species closely related to the producing strain [3,7].

We suggest that the mechanism underlying the variation in the antagonistic effects of toxins like bacteriocins is caused by intraspecific resource competition. We expect that the ability of these toxins to remove competitors, and so free up resources, would evolve to be maximal when resource competition is strongest among genetically distinct individuals. The logic behind this is straightforward. Toxin production should not be favoured when competing with genetically identical clones because there is no fitness benefit to production. As genetic distance increases, however, so too does phenotypic and ecological divergence [8,9], and by extension resource competition. Toxin production is therefore wasted when competing against genetically very divergent strains because there is little resource competition. In other words, toxin production becomes costly because its benefits are diluted by the fact that the producer and target strain do not compete with each other.

This interpretation leads to the prediction that the strength of antagonism should peak at intermediate genetic distance. To test this prediction we studied the interaction between two producer strains that produce a multitude of bacteriocins and a range of sensitive 'victim' strains of varying genetic distance to the producers. Specifically, we measured the ability of anticompetitor toxins produced by two laboratory strains of *Pseudomonas aeruginosa*, PA01 and PA14, to kill or inhibit 55 clinical strains of *P. aeruginosa *isolated from the lungs of cystic fibrosis (CF) patients. Natural communities of microbes associated with chronic infections such as colonization of the cystic fibrosis lung are often highly diverse [10-13]. We also measured the degree of ecological similarity among strains, using commercially available BIOLOG plates that contain 95 different carbon substrates, and show that ecological similarity can decrease with genetic distance. This result is consistent with the idea that toxin production is not favoured among genetically divergent strains because of a lack of resource competition.

### Pyocins and *Pseudomonas aeruginosa*

*P. aeruginosa *produces a wide variety of toxins and among the most interesting, in part because they are known to be highly specific in their action, are bacteriocins called pyocins. They are costly to produce because they are released by cell lysis of a fraction of the producer population. Pyocins are proteinaceous compounds that are classified into three groups (R-, F-, and S-type), with multiple sub-types within each group that attach to different potential receptors in target strains [5,14,15]. PA01 is known to produce all three pyocins while PA14 produces only R- and F-type pyocins [4]. Genes coding for production of all pyocins are located on the chromosome and are clustered with genes coding for resistance to the same pyocins. Genomic studies have suggested the presence of more pyocins [16-19], both from the S- and R-types. In addition, a recently developed genome-mining tool for bacteriocins has revealed the general existence of yet to be characterized bacteriocins in several bacterial species [20]. Other toxins produced by *P. aeruginosa *include virulence factors such as exotoxin A, PCN and Y as well as membrane vesicles [21-23].

The clinical strains in our study come from a multi-centre Canadian study of the epidemiology of chronic *P. aeruginosa *infections of CF patients [24], see Methods. Chronic infection with P. aeruginosa occurs in 60-70% of Canadian adults with CF [25]. After confirmation using standard techniques that the isolates were P. aeruginosa (Methods), genetic distance among all strains was estimated by comparing banding patterns of a full genome digest using pulsed field gel electrophoresis, PFGE [26-30]. We also confirmed that genetic distance correlates with the degree of overlap in resource use, measured by the ability of strains to metabolize 95 different carbon substrates found on commercially available Biolog plates.

## Results and discussion

**Figure 1:**
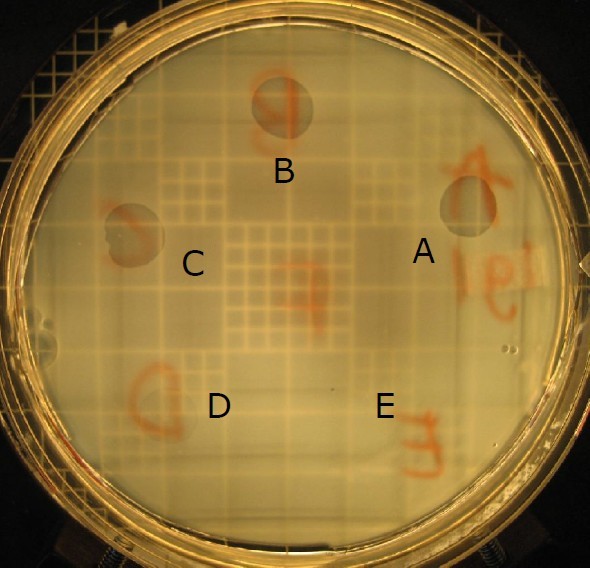
**Inhibition assay**. Lawn of a *Pseudomonas aeruginosa *natural isolate growing on the surface of an agar plate. Spots of pyocin containing cell free extract from a laboratory strain of *P. aeruginosa *PA01 were applied on the lawn at different dilutions. The formation of clear zones is indicative of killing of the clinical isolate. The highest dilution of cell free extract (thus containing the lowest concentration of toxin) that inhibits the clinical isolate is a measure of potency of the toxin. The inhibition score is the inverse of the highest dilution that inhibits growth of the clinical isolate. In this example, the spot marked A is non-diluted cell free extract; spots B to F are serial 3-fold dilutions. The inverse of the dilution factor of dilution D would be the inhibition score.

**Figure 2:**
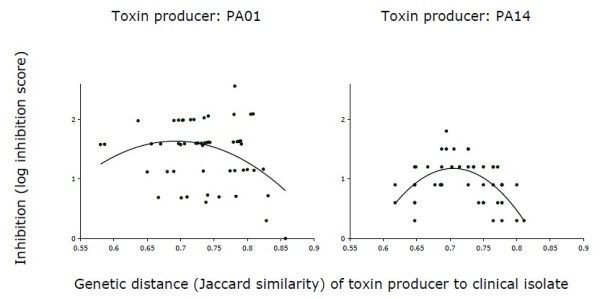
**Inhibition by toxin containing cell free extract**. Inhibition of clinical isolates by toxins in cell free extract collected from laboratory strains PA01 and PA14 as a function of genetic distance (Jaccard similarity) between toxin producer and clinical isolate. A unimodal non-linear relationship peaking at intermediate Jaccard distance give best fit to the data (solid lines), better than a linear fit, see text and Table 1.

We measured the level of inhibition by anticompetitor toxins by spotting a dilution series of a cell free extract collected from 48 h old *P. aeruginosa *PA01 or PA14 culture onto a lawn of one of 55 different clinical isolates growing on a solid surface. The natural isolates differ in their genetic distance to the producing strain; genetic distance is quantified using full genome digests. The lowest concentration of cell free extract that gave rise to inhibition of the clinical isolate was used to calculate the inhibition score (Figure 1) [14,15,31,32]. Figure 2 depicts the level of inhibition by both PA01 and PA14 as a function of genetic distance of toxin producing strain to the clinical isolates.

Our results lend strong support to the idea that toxins are most effective when active against genotypes of intermediate genetic distance relative to the focal strain. The relationship between inhibition and genetic distance is unimodal, peaking at intermediate genetic distance for both toxin producers PA01 and PA14. This result is confirmed more formally by noting that a quadratic model with an internal maximum is a better descriptor of the data than a linear model (Table 1; in the linear regressions, the linear term is not significant), by the lower AIC (Aikake's Information Criterion) values for the quadratic models than the linear models (Table 1) and by an F-ratio test asking if adding the quadratic term provides a significantly better fit than the linear model (PA01, F_1,48 _= 5.96, *P *= 0.018; PA14, F_1,42 _= 17.56, *P *= 0.00014). We also tested for the existence of an internal maximum in the data using a Mitchell-Olds and Shaw (MOS) test (as implemented in the R package vegan) following Mittelbach et al. (2001) [33]. This approach tests the null hypothesis that a quadratic function, fitted to the data, has no stationary point (either a maximum or minimum) within the range provided. Our results reject this null hypothesis for both PA01 and PA14 at the *P <*0.1 level (PA01: *P *= 0.072; PA14: *P *= 0.0006), the same criterion used in Mittelbach et al. (2001) [33]. Since the sign of the quadratic coefficient for both producer strains was negative, the results of the MOS test indicate the presence of a statistically significant 'hump' within the range of genetic distances examined. The evidence for an internal hump is somewhat weaker for PA01 than PA14 but we note that our test is conservative, as we have not included data on the effectiveness of either strain at inhibiting itself. As both of these values are zero (see Methods), including these values would produce a much more pronounced hump.

**Table 1:** Linear and quadratic regressions of inhibition of clinical isolates by sterile (non heat treated) cell free extract of PA01 and PA14 cultures as function of genetic distance (Figure 2)

Source	df	Value	St Error	*t*	P-value	Multiple R^2^	AIC
**PA01 **Linear model					0.072	0.059	90.91
Intercept	1	3.27	0.969	3.38	0.0014		
Linear term	1	-2.41	1.31	-1.84	0.072		
Residual SE	53		0.55				

**PA01 **Quadratic model					0.010	0.160	86.94
Intercept	1	-17.00	8.81	-2.08	0.043		
Linear term	1	53.94	22.61	2.38	0.021		
Quadratic term	1	-38.89	15.58	-2.50	0.016		
Residual SE	52		0.53				

**PA14 **Linear model					0.15	0.044	39.80
Intercept	1	1.99	0.71	2.81	0.0072		
Linear term	1	-1.45	0.98	-1.48	0.15		
Residual SE	47		0.36				

**PA14 **Quadratic model					< 0.0001	0.345	26.08
Intercept	1	-37.51	8.62	-4.35	0.0001		
Linear term	1	109.8	24.23	4.53	< 0.0001		
Quadratic term	1	-77.88	16.95	-4.59	< 0.0001		
Residual SE	46		0.30				

To verify that genetic distance correlates with resource use, we measured the metabolic similarity of toxin producing strains to the clinical isolates using Biolog plates (see Methods). Metabolic profiles become more divergent with increasing genetic distance, as expected, reflected in the significantly negative linear relationship observed between Jaccard distance and metabolic correlation between pairs of strains (PA01: slope ± standard error = -0.493 ± 0.213; multiple R^2 ^= 0.098, *t*_,49 _= -2.312, *P *= 0.025; PA14: slope ± standard error = -0.644 ± 0.208, multiple R^2 ^= 0.164, *t*_49 _= -3.104, P = 0.0032). These results lend support to the idea that genetic distance is linked to ecological divergence. It is further notable that inhibition score peaked at intermediate metabolic similarities for both PA01 and PA14 but was statistically significant only for PA14 (see Additional file 1: Table S1 and Additional file 2: Figure S1; F-ratio test on the fitting of the quadratic term, PA01: F_1,48 _= 0.176, *P *= 0.68; PA14: F_1,42 _= 7.00, *P *= 0.011).

It is not immediately obvious why we detected a significant quadratic relationship between inhibition score and metabolic similarity in one strain but not the other. One possibility is that the Biolog plates we used here, which provide profiles on carbon substrate metabolism, represent one of many possible dimensions along which ecological divergence can proceed. Under this interpretation, metabolic divergence in carbon substrate use may reflect a correlated response to divergence in other ecologically important factors such as ability to grow as a biofilm, resistance to various stressors like pH, temperature, or salinity, and possibly even predation, that we did not measure. Thus we would still expect to see some relationship between metabolic similarity and genetic distance, as we did for PA01, even if this is not the sole target of ecological divergence. There are any number of other differences between PA01 and PA14 that could be responsible for this difference. PA14 has a slightly larger genome than PA01 (6.5 Mbp and 6.3 Mbp, respectively) and contains a number of unique 'pathogenicity islands' that are thought to be associated with a generally increased level of virulence in most hosts [34]. It also is thought to produce only R- and F-type pyocins, whereas PA01 produces all three types (R, F, and S) [4]. It is notable that S-pyocins differ from both R- and F-pyocins in that they are oligopeptides whereas R- and F-pyocins are both phage-like structures. Why or how the differences in genome content, size, or pyocin identity affects the relationship between inhibition score and metabolic similarity remains an open question, however.

**Figure 3:**
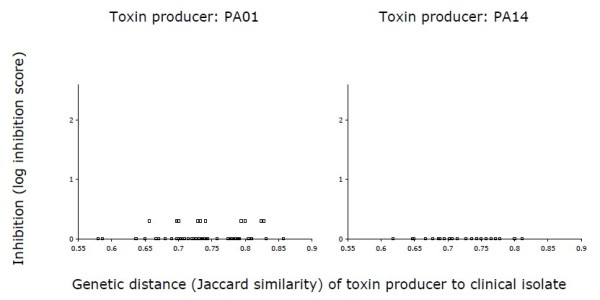
**Inhibition by heat treated cell free extract**. Inhibition of clinical isolates by heat treated cell free extract collected from laboratory strains PA01 and PA14 as a function of genetic distance (Jaccard similarity). No regression gave a significant fit to the data.

What agents are responsible for killing in our experiments? Bacteriophage were clearly not responsible. If bacteriophage were causing the inhibition of clinical isolates, they would be able to amplify themselves in an exponential culture of the same clinical isolate. This was not the case (see Methods). Three lines of evidence suggest, rather, that toxic compounds such as pyocins or exotoxins excreted by PA01 and PA14 are the main killing agent. The first is that PA01 and PA14 are not killed by their own supernatant. Such a result is consistent with the idea that the toxins are pyocins, as pyocin production involves specific immunity genes that confer resistance by preventing lysis in non-producing kin [4,5,35,36], although it does not rule out the possibility that other toxins with similar immunity properties are also involved. If killing were associated with a non-specific toxic compound such as some waste product, we would have expected both producer strains to be susceptible to killing and killing would most likely also not depend on genetic or metabolic similarity. Second, repeating the inhibition assay with heat-treated supernatant eliminates killing (Figure 3; both linear and quadratic regressions are non-significant), providing strong support for the idea that the killing compounds are proteins. Third, and most interestingly, inhibition by PA01 is stronger, on average, than that by PA14 (mean log inhibition score for PA01 = 1.51; mean log inhibition score for PA14 = 0.95; t-test, *t*_93 _= 6.05, *P *< 0.0001), a result that is likely due to the fact that PA01 produces a larger array of pyocins than PA14, including S-type pyocins [4].

Although these three lines of evidence point suggestively to pyocins as being the main killing agent, we have not conducted an explicit test of this hypothesis by, for example, repeating our assays with pyocin knock-out strains. Although it may be possible to conduct such a test by focusing on the prtR/N regulator, which is thought to be a global regulator of known pyocins [4,5], it is not clear that such a test would be conclusive since a number of the pyocins in both PA01 and PA14 have yet to be isolated [18,19] and there may exist other exotoxins that behave in similar ways to pyocins. Note also that knowing the mechanism of killing, while of obvious interest, is in many ways of secondary importance to the observation that the effectiveness of killing depends in a regular way on genetic distance, at least in the strains we have studied here.

Our main result is that the strength of antagonistic interactions peak at intermediate genetic distance. This pattern is strikingly similar to that expected from theoretical [37] and experimental [38,39] kin selection models for selection using mixed populations of two strains at various ratios to adjust relatedness and considering one bacteriocin and one immunity protein. These models have emphasized how the cost of bacteriocin production is affected by the social environment: bacteriocin production is not favored when producers are both common, because the majority of competitors are kin and so immune to the bacteriocin, and rare, because there are now too few kin to enjoy the benefits of the extra resources. This is clearly not an appropriate interpretation of our results because we did not manipulate the frequency of producers and non-producers in our experimental system to adjust relatedness, as Inglis et al. [38] have done using degree of kinship as a measure of relatedness.

Rather, our results provide some evidence consistent with the idea that ecological divergence may be important in mediating social interactions. It is notable that the explanation for the ineffectiveness of toxins at inhibiting closely related genotypes (i.e. short genetic distance) in our experiment is likely similar to that in kin selection models: they share a degree of immunity to each other's toxins. However, the ineffectiveness of toxins against distantly related genotypes in our system is probably not directly tied to kin selection. Because increasing genetic divergence is accompanied by reduced overlap in resource use, distantly related genotypes are unlikely to compete for similar resources and so the resources liberated through antagonism are therefore unlikely to benefit the producer [8,40]. The production of antagonistic traits such as bacteriocins in this situation is therefore likely to be costly and so selection should lead to decreased levels of antagonism. Our observation of decreased antagonism among distantly related strains, at least for PA14, is consistent with this interpretation.

This interpretation is in accordance with the idea that the evolution of specific antagonism against conspecifics, as is often observed for bacteriocins, could evolve as a direct response to intraspecific resource competition and is consistent with models describing how fitness varies as a function of the strength of resource competition between types: the strength of resource competition, and so the loss of fitness, is highest when resource overlap is intermediate [41]. To the extent that the opportunity or intensity of resource competition is enhanced through the physical proximity of co-occurring strains in a given habitat [42], such as a CF lung, then this may further promote the evolution of antagonistic interactions such as those mediated by bacteriocins. It remains to be seen whether our results are specific to the strains we used in this study or whether they apply more broadly to non-CF strains of *P. aeruginosa *or other species. This will be an important avenue for future research.

It is not possible with our data to distinguish the specific mechanisms causing variation in toxin susceptibility. If bacteriocins are indeed responsible for killing, then one possibility is that selection targets the total amount of bacteriocin production or the efficiency with which bacteriocins inhibit or kill their victims. It is also possible that the target of selection is the number of receptor sites for bacteriocins in target strains. Deciding among these alternatives requires follow-up experiments that focus on finding the evolutionary origin of bacteriocins using direct competition experiments of producer stains and several target strains to ask under what conditions and by what mechanism bacteriocins aid producer populations to invade populations of sensitive strains [43]. These experiments would however be very elaborate since the effect of many complicating factors such as frequency dependence, cross-feeding, the viscosity of the environment and exact costs of producing bacteriocins would have to be determined for the interaction of the producer and each target strain. It is even possible that the high specificity of bacteriocins results from their having evolved initially as a by-product of selection for fertility-recognition systems such as conjugation that were later co-opted for use as bacteriocidal agents [49]. Investigating the relationship between bacteriocin diversity and conjugation frequency or recombination could help shed some light on this issue.

Our results have important implications for understanding of the dynamics of infection in clinical settings. We have firmly established that toxic compounds with high specificity mediate bacterial interactions as antagonistic agents, for instance in structuring pathogen populations in patients with a mixed *P. aeruginosa *infection [12]. Social evolution theory predicts that selection for antagonism among pathogenic strains should be accompanied by reduced virulence to the host. The consequences of *P. aeruginosa *infection on patient morbidity and mortality may therefore depend to some extent on the particular strains present. Furthermore, it is notable that recent research on CF patients from Ontario suggests that 25% of Ontario patients who are infected with *P. aeruginosa *are infected with one of two predominant epidemic strains. It may be that the predominance of these epidemic strains is due to the production of specific antagonistic agents such as pyocins [13]. This is an intriguing hypothesis that awaits further testing. As a start, we have confirmed that three of our clinical isolates produce toxic substances that kill or inhibit other clinical isolates (data not shown). Thus the antagonistic interactions we have studied here do happen among clinical isolates and are not just the consequence of using strains PA01 and PA14 as producers in our study [13].

Understanding the way toxins such as pyocins kill *P. aeruginosa *strains, and how this is modulated by genetic relatedness, may also provide insight into the development of novel therapeutic interventions, for example by evolving pyocins specifically against strains that predominate in infections. They can thus be considered designer drugs [7,23,44,45] and will be a much more direct agent to treatment of the disease than the current practice of using broad spectrum antibiotics against which wide spread resistance exists [46]. Interestingly, pyocins are not new in a clinical setting: it has been shown that pyocins slow down the development of several forms of cancer in mammalian cells [47]. Also, membrane vesicles produced by *P. aeruginosa *have been suggested as novel therapeutic agents [23]. However they may be even more effective when used in a targeted way against known infections. The similarity between strains can then be used as a predictor of the intensity of the antagonistic interaction and thus the effectiveness of the pyocin.

## Conclusions

Using clinical and laboratory strains of *Pseudomonas aeruginosa*, we found that the level of antagonism between toxin producing and target strains is maximal at intermediate genetic and metabolic similarity between producer and target strain. We explained this result in the context of resource competition: resource competition is expected to be maximal for strains that are not your kin but also not completely unrelated since those strains do not share the same need for resources and are less likely to be a competitor. Our results suggest that the importance of antagonism and perhaps other social interactions between bacteria are modulated by the strength of resource competition.

## Methods

### Bacterial strains and culture conditions

We used standard laboratory strains *Pseudomonas aeruginosa *strains PA01 and PA14 and 55 natural *P. aeruginosa *isolates collected from cystic fibrosis patients. Infection with *P. aeruginosa *is associated with increased morbidity and mortality for CF patients, irrespective of lung function. Both population-based and case-control studies have demonstrated that infection with *P. aeruginosa *bacteria appears to be an independent prognostic factor that carries with it an increased risk of death [38,40]. Strains used here were isolated from sputum samples obtained from multiple patients all of whom had chronic endobronchial infections. Clinical *P. aeruginosa *isolates were collected between September 2005 and June 2008 from adult patients with confirmed cystic fibrosis who attended one of the seven Ontario adult cystic fibrosis clinics or who attended smaller outreach clinics [24]. These 7 clinics provide secondary and tertiary care to more than 97% of all CF patients in Ontario. Patients were included in the study if they were ≥ 18 years of age, able to spontaneously produce sputum, and if they had a confirmed diagnosis of cystic fibrosis (a sweat chloride value higher than 60 mmol/litre and/or 2 disease-causing mutations). The research ethics board (The Ottawa Hospital Research Ethics Board) of all the participating centers approved the study, and all participants provided written informed consent. Patients provided sputum samples which were couriered on ice to the central laboratory in Ottawa. To detect *P. aeruginosa *and other bacterial pathogens, sputum was plated onto the following selective and nonselective media: Columbia blood agar plate (PML), MacConkey agar plate (PML), and *Pseudomonas aeruginosa *selective agar plate (Oxoid). Plates were incubated at 35°C for 48 hours and *P. aeruginosa *colonies were identified by oxidase testing, TSI, arginine and growth at 42°C. If *P. aeruginosa *was isolated, then two distinct *P. aeruginosa *colony morphotypes from each sputum were worked up for molecular typing, and five *P. aeruginosa *isolates derived from each sputum were frozen at -70°C.

To prepare for inhibition assays, strains were streaked from frozen on Pseudomonas Isolation Agar (Difco). Single colonies were used to inoculate liquid LB (bacto-tryptone 10 g, yeast extract 5 g, NaCl 10 g, dH_2_O 1000 ml) and incubated under aerobic shaken conditions (150 rpm) at 37°C for 24 to 48 h to yield dense cultures.

### Estimation of genetic distance

Genetic distance was estimated by comparing molecular genotypes of each *P. aeruginosa *isolate generated through pulsed-field gel electrophoresis (PFGE). PFGE is a well-accepted method [26,30] that differs from multi-locus sequence typing (MLST)-based approaches in that it includes the entire genome rather than just seven housekeeping genes. MLST profiling of our strains using seven housekeeping genes showed high similarity for those genes; what we would expect since they were all classified as *P. aeruginosa*. Studies [27] have shown that PFGE is more accurate when typing very closely related strains from the same species. To generate PFGE profiles, genomic DNA was prepared by a modification of a previously described method [48]. *P. aeruginosa *isolates were grown overnight at 37°C on Tryptone Soya Agar plates containing 5% sheep's blood. Colonies were suspended in buffer (1 M NaCl, 10 mM Tris-HCl [pH 7.6]) and 70 μL of the suspension was mixed with an equal amount of 1.6% low melt agarose (Cambrex, East Rutherford, NJ). This mixture was pipetted into a plug mold (Bio-Rad, Hercules, CA) and allowed to solidify at room temperature. Plugs were added to plug lysis solution (1 M NaCl, 100 mM EDTA [pH 7.5], 0.5% Brij-58, 0.5% Sarcosyl, 0.2% Deoxycholate, 6 mM Tris-HCl [pH 7.6], 1 mg/mL Lysozyme powder, 20 μg/ml RNase) and incubated for 4 h at 37°C with shaking. Plugs were then placed in Proteinase K solution (0.5 M EDTA [pH 9-9.5], 1% Sarcosyl, 50 μg/ml Proteinase K) and incubated overnight at 50°C with shaking. Plugs were washed 3-4 times with TE buffer (10 mM Tris-HCl [pH 7.5], 0.1 mM EDTA [pH 7.5]) at 37°C and then stored at 4°C. DNA in a 2-3 mm piece of the gel plug was restricted using 20 U SpeI (New England Biolabs, Ipswich, MA) in a reaction volume of 0.2 mL at 37°C. The digestion products were melted and electrophoresis was performed on a 1.0% agarose gel, in 0.5X TBE (VWR International Ltd, Mississauga, ON), using a CHEF DR III apparatus (Bio-Rad, Hercules, CA). Electrophoresis conditions were as follows: 20 h at 6 V/cm with switch times of 5 s to 45 s with a linear ramping factor. Using the ladder, all banding patterns were inspected for the presence/absence of a visible band at 51 locations. These presence/absence data were used to calculate the genetic distance by calculating the Jaccard similarity (Jaccard distance equals 1- Jaccard similarity) of natural isolates to both laboratory strains PA01 and PA14: $J^{\prime }=\frac{M_{11}}{M_{01}+M_{10}+M_{11}}$ where M_ij _represents the total number of positions where bands are present (*i = j = *1), or when one strain or the other possesses a band (*i ≠ j*). Other measures of similarity such as the Hamming distance, Dice coefficient and correlation coefficient gave similar qualitative results. We used R software (version 2.6.1) to calculate distance measures and for all statistical analyses.

### Estimation of metabolic similarity

Resource use was measured using BIOLOG GN2 plates that consist of different wells with a total of 95 different carbon sources. All 55 clinical isolates and strains *P. aeruginosa *PA01 and PA14 were grown up in liquid LB medium. From a dense stationary phase culture, 20 μl was added to 20 ml of a minimal salts medium (Na_2_HPO_4 _6.7 g, KH_2_PO_4 _3 g, NaCl 0.5 g, NH_4_Cl 1.0 g, 1000 ml dH_2_O) which was used to inoculate the Biolog plates after a 2 h starvation period. For clinical isolates, 1 Biolog plate was used, for *P. aeruginosa *PA01 and PA14 three replicate plates were used. Right after inoculation and after 48 h of incubation at 37°C, the OD (590 nm) was measured of all wells. The difference in OD at the two time points is a measure of how well a given strain is able to use a given resource. To quantify the metabolic similarity, we calculated the correlation coefficient between the OD values of the different strains.

### Inhibition assays

The strains *P. aeruginosa *PA01 and PA14 were assayed for their ability to produce toxic compounds that can inhibit the natural *P. aeruginosa *isolates collected from CF patients using a method adopted from Jacob 1954 [14]. Bacterial cells of dense LB cultures (48 h old) of PA01 and PA14 were spun down in a centrifuge. Supernatant was collected and filtered (0.20 μm) and serially diluted in minimal salts medium [14]. For the assay asking if proteinaceous compounds are responsible for killing, the sterile supernatant was heated for 15 s at 100°C. 0.5 ml of a dense culture of a natural isolate was added to 3 ml of molten semi solid LB (bacto-tryptone 10 g, yeast extract 5 g, NaCl 10 g, agar 7 g, 1000 ml dH_2_O) and poured out over the surface of a Petri dish containing minimal salts agar (as described above with 10 g of agar added) as an overlay. A dilution series of either non-heat treated or heat treated sterile supernatant was spotted on top of the layer of natural isolate, up to 11 spots of 15 μl were spotted on a single Petri dish with overlay. Cultures were incubated for 48 h at 37°C. The inverse of the highest dilution of sterile supernatant giving rise to inhibition was defined as the inhibition score, which is effectively a measure of the minimal inhibitory concentration (MIC). The inhibition scores were log transformed prior to analysis. No inhibition was observed when spotting sterile PA01or PA14 supernatant on top of an overlay with the same strain.

To exclude the possibility that bacteriophage are responsible for the observed inhibition, we performed a one-step growth assay as follows. The zones of inhibition formed on overlays of clinical isolates were transferred to an exponential liquid culture of growing cells of the same clinical isolate. After 24 h, cell free extract was prepared and spotted onto a layer of the clinical isolate. A resulting clear zone of inhibition would be indicative of the presence of bacteriophage because a 24 h liquid culture of clinical isolate should contain even more bacteriophage than the initial culture since bacteriophage are able to reproduce with the appropriate host cells present. We found no evidence for the presence of bacteriophage.

## Authors' contributions

Conceived the study: SES, SDA, RK; designed protocols: SES, JD, RD; performed experiments: SES, JD, RD; analyzed data: SES, JD, RK; collected clinical isolates and generated PFGE profiles: SDA; Wrote the paper: SES, RK. All authors commented on and approved the final manuscript.

## Supplementary Material

Additional file 1 **Table S1**. Inhibition of clinical isolates by toxins in cell free extract collected from laboratory strains PA01 and PA14 as a function of metabolic similarity (correlation coefficient) between toxin producer and clinical isolate based on BIOLOG profiles. A unimodal non-linear relationship peaking at intermediate metabolic similarity give best fit to the data for producer PA14 (solid lines), better than a linear fit; for PA01 no such relationship was found. See text and Supplemental Table. Click here for file

Additional file 2 **Figure S1**. Inhibition of clinical isolates by toxins in cell free extract collected from laboratory strains PA01 and PA14 as a function of metabolic similarity (correlation coefficient) between toxin producer and clinical isolate based on BIOLOG profiles. A unimodal non-linear relationship peaking at intermediate metabolic similarity give best fit to the data for producer PA14 (solid lines), better than a linear fit; for PA01 no such relationship was found. See text and Supplemental Table. Click here for file
