# Supplementary material for: Antagonistic interactions peak at intermediate genetic distance in clinical and laboratory strains of Pseudomonas aeruginosa
Source: BMC Microbiol. 2012 Mar 22;12:40. doi: 10.1186/1471-2180-12-40 (PMC3391984; doi:10.1186/1471-2180-12-40)
Supplement: Additional file 1 — Table S1. Inhibition of clinical isolates by toxins in cell free extract collected from laboratory strains PA01 and PA14 as a function of metabolic similarity (correlation coefficient) between toxin producer and clinical isolate based on BIOLOG profiles. A unimodal non-linear relationship peaking at intermediate metabolic similarity give best fit to the data for producer PA14 (solid lines), better than a linear fit; for PA01 no such relationship was found. See text and Supplemental Table. [file 1471-2180-12-40-S1.DOC]

## Supplemental Table.

*Linear and quadratic regressions of inhibition of clinical isolates by sterile (non heat treated) cell free extract of PA01 and PA14 cultures as function of metabolic similarity (see Methods).*

| ***Source*** | ***df*** | ***Value*** | ***St Error*** | ***t*** | ***P-value*** | ***Multiple R^2^*** | ***AIC*** |
| --- | --- | --- | --- | --- | --- | --- | --- |
| ***PA01*** *Linear model* |  |  |  |  | *0.33* | *0.019* | *92.48* |
| *Intercept* | *1* | *2.16* | *0.696* | *3.10* | *0.0032* |  |  |
| *Linear term* | *1* | *-0.87* | *0.89* | *-0.98* | *0.33* |  |  |
| *Residual SE* | *49* |  | *0.576* |  |  |  |  |
|  |  |  |  |  |  |  |  |
| ***PA01*** *Quadratic model* |  |  |  |  | *0.57* | *0.022* | *94.29* |
| *Intercept* | *1* | *0.299* | *4.476* | *0.0668* | *0.95* |  |  |
| *Linear term* | *1* | *4.10* | *11.88* | *0.35* | *0.73* |  |  |
| *Quadratic term* | *1* | *-3.28* | *7.81* | *-0.42* | *0.67* |  |  |
| *Residual SE* | *48* |  | *0.581* |  |  |  |  |
|  |  |  |  |  |  |  |  |
| ***PA14*** *Linear model* |  |  |  |  | *0.45* | *0.013* | *40.34* |
| *Intercept* | *1* | *0.55* | *0.549* | *1.00* | *0.32* |  |  |
| *Linear term* | *1* | *0.53* | *0.706* | *0.727* | *0.45* |  |  |
| *Residual SE* | *43* |  | *0.362* |  |  |  |  |
|  |  |  |  |  |  |  |  |
| ***PA14*** *Quadratic model* |  |  |  |  | *0.030* | *0.15* | *35.41* |
| *Intercept* | *1* | *-9.21* | *3.72* | *-2.47* | *0.017* |  |  |
| *Linear term* | *1* | *26.46* | *9.82* | *2.69* | *0.010* |  |  |
| *Quadratic term* | *1* | *-17.04* | *6.44* | *-2.65* | *0.011* |  |  |
| *Residual SE* | *42* |  | *0.340* |  |  |  |  |
