# Supplementary figures and images for: Antagonistic interactions peak at intermediate genetic distance in clinical and laboratory strains of Pseudomonas aeruginosa
Source: BMC Microbiol. 2012 Mar 22;12:40. doi: 10.1186/1471-2180-12-40 (PMC3391984; doi:10.1186/1471-2180-12-40)

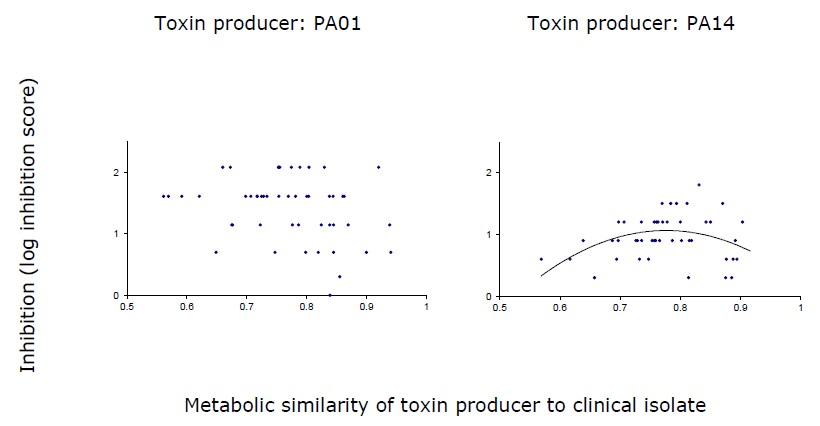

Supplement: Additional file 2 — Figure S1. Inhibition of clinical isolates by toxins in cell free extract collected from laboratory strains PA01 and PA14 as a function of metabolic similarity (correlation coefficient) between toxin producer and clinical isolate based on BIOLOG profiles. A unimodal non-linear relationship peaking at intermediate metabolic similarity give best fit to the data for producer PA14 (solid lines), better than a linear fit; for PA01 no such relationship was found. See text and Supplemental Table. [file 1471-2180-12-40-S2.JPEG]
